# Application of ultrasound-guided percutaneous nephrostomy in the treatment of a solitary kidney with hydronephrosis due to renal tuberculosis

**DOI:** 10.1007/s00261-023-04068-9

**Published:** 2023-11-08

**Authors:** Bingsong Zhang, Lei Li, Gengchen Zhang, Jinshan Wang, Bingsheng Cao, Zhuo Li

**Affiliations:** Department of Ultrasound, Eighth Medical Center of Chinese PLA General Hospital, Beijing, China

**Keywords:** Interventional ultrasound, Renal tuberculosis, Hydronephrosis, Nephrostomy

## Abstract

**Purpose:**

To investigate the value of ultrasound-guided percutaneous nephrostomy and nephrostomy tube replacement for treating a solitary kidney with hydronephrosis due to renal tuberculosis.

**Methods:**

Clinical data of patients with a solitary kidney with hydronephrosis caused by renal tuberculosis who underwent ultrasound-guided percutaneous nephrostomy in our hospital from January 2011 to December 2022 were retrospectively analyzed. The associated success rate and complications were statistically analyzed, pre- and post-catheterization changes in serum creatinine and blood urea nitrogen levels were compared, success rate and complications of nephrostomy tube replacement in patients with long-term catheterization were statistically analyzed, and the impact of long-term catheterization on patient life was investigated.

**Results:**

Overall, 32 patients aged 17–75 years (average age: 44.1 ± 16.9 years) underwent ultrasound-guided percutaneous nephrostomy. Sixty-three punctures were performed; the puncture success rate was 100%. The levels of serum creatinine and blood urea nitrogen of patients decreased after catheterization, and the differences between the pre-catheterization and post-catheterization were significant (*P* < 0.05). There were 1, 3, and 12 cases of serious, minor, and fistula-related complications, respectively. The mean duration of the indwelling catheter was 56.7 ± 36.2 (range, 13–120) months. The number of nephrostomy tube replacements was 344 times, and the success rate was 100%. All patients could take care of the puncture point by themselves.

**Conclusion:**

Ultrasound-guided percutaneous nephrostomy and nephrostomy tube replacement have a high success rate and few complications, which can improve the renal function of patients. It is of great value for treating a solitary kidney with hydronephrosis caused by renal tuberculosis.

## Introduction

Genitourinary tuberculosis is the second most common type of extrapulmonary tuberculosis after lymphatic tuberculosis, among which renal tuberculosis is the most common [1], and its incidence has shown an increasing trend in recent years [2, 3]. Due to the lack of specific manifestations in the early stage and the difficulty of diagnosis [4], in most patients, renal tuberculosis is diagnosed in the middle and late clinical stages. At present, anti-tuberculosis drugs are used to manage early renal tuberculosis, whereas intermediate and advanced stage of renal tuberculosis are mainly treated by surgery [5]. For patients with renal tuberculosis who have normal renal function and severe urinary tract stenosis, urethroplasty or internal stenting is an option. On the other hand, for those in whom urethroplasty cannot be performed or stenting fails, percutaneous nephrostomy drainage is an optional treatment. In this study, we investigated the effectiveness and complications of ultrasound-guided percutaneous nephrostomy for treating patients with renal tuberculosis who underwent unilateral nephrectomy and developed contralateral ureteral obstruction with hydronephrosis with an aim to explore the application of ultrasound-guided percutaneous nephrostomy in the management and treatment of such patients.

## Materials and methods

### Patients

Patients with hydronephrosis in a solitary kidney and renal tuberculosis who were admitted to the Tuberculosis Department of our hospital from January 2011 to December 2022 were enrolled. The inclusion criteria were as follows: (1) renal tuberculosis confirmed by pathology or bacteriology testing in patients who underwent unilateral nephrectomy and developed hydronephrosis in the contralateral kidney; (2) imaging-based diagnosis of ureteral obstruction, failure of urethroplasty or ureteral stent implantation at admission; and (3) patients who underwent percutaneous nephrostomy more than 12 months and were receiving long-term follow-up. The following patients were excluded: (1) patients with severe cardiopulmonary dysfunction who could not tolerate surgery; (2) patients with a severe infection and a malignant tumor; (3) patients with abnormal coagulation function; (4) patients with mental illness who are unable to cooperate with treatment; and (5) patients with renal insufficiency who had undergone dialysis treatment. All patients signed informed consent before undergoing ultrasound-guided catheter drainage. Overall, 32 patients were enrolled, including 20 men and 12 women (mean age: 44.1 ± 16.9 years, range: 17–75 years).

### Instruments and ultrasound-guided percutaneous nephrostomy

#### Instrument

The Aloka SSD α10 Prosound diagnostic system with a UST-9133 micro-convex probe (frequency 3–5 MHz) was used.

#### Method of ultrasound-guided percutaneous nephrostomy

All patients underwent routine urinary ultrasound examination to determine the size and shape of the kidney; blood flow signal; degrees of renal pelvis separation, ureteral dilatation, ureteral wall thickening, and bladder wall thickening; and bladder size.

**Fig. 1:**
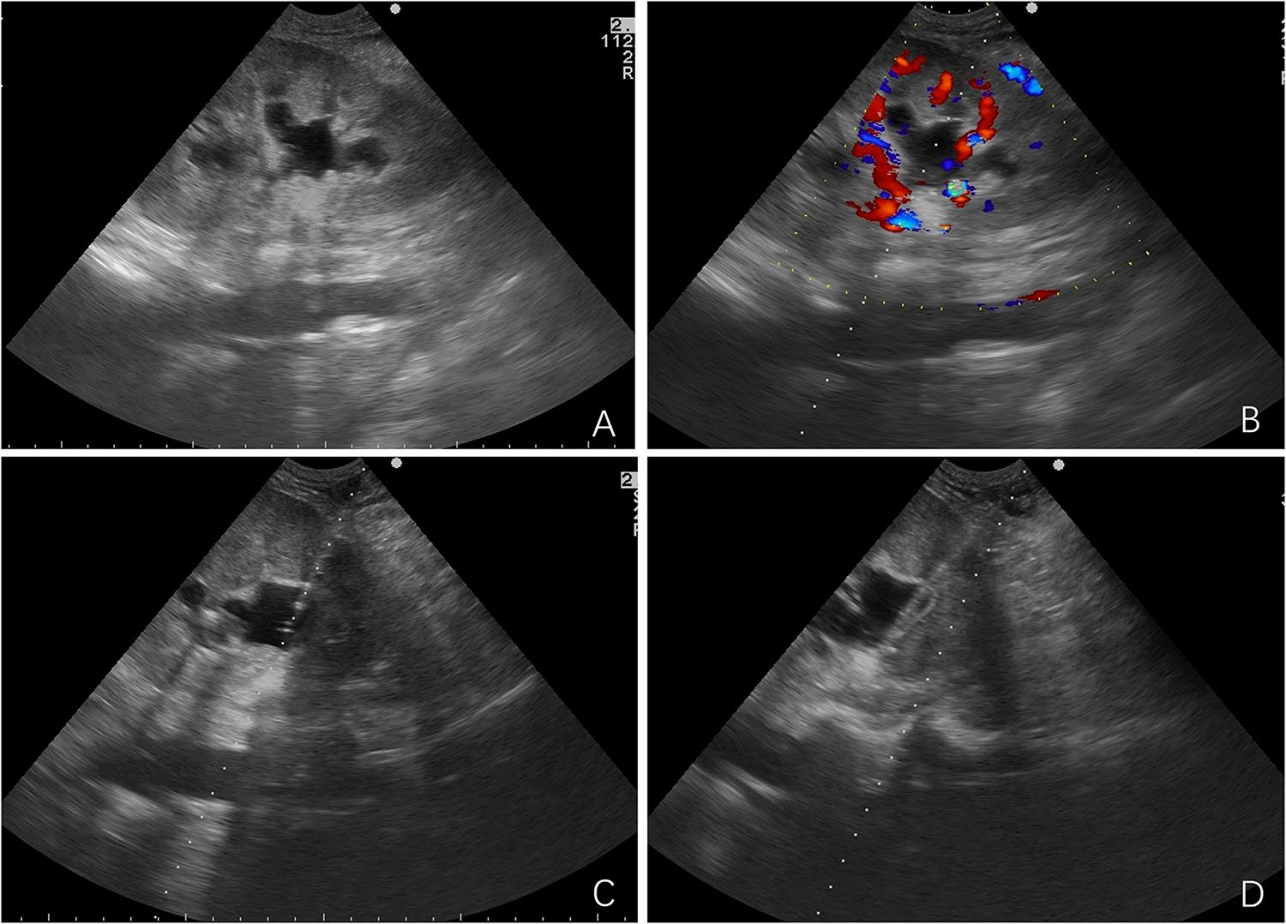
Method of ultrasound-guided percutaneous nephrostomy: **A** Gray-scale ultrasound showed hydronephrosis; **B** Color Doppler ultrasound was used to plan the puncture path; **C** Insertion of the drainage tube kit by puncture; **D** The drainage tube was placed in the renal pelvis

**Fig. 2:**
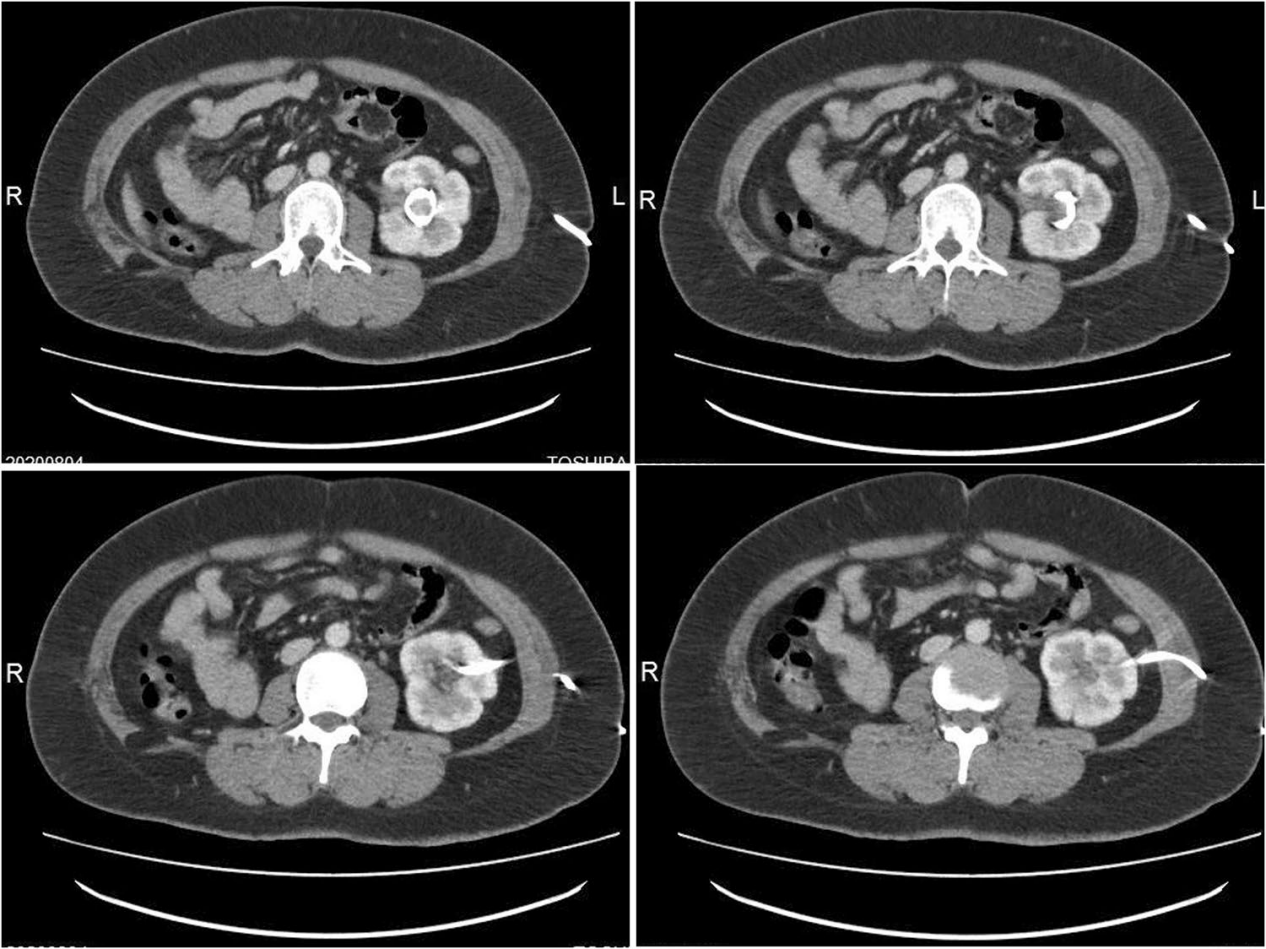
Abdominal CT images showed that the patient’s right kidney was absent, and a drainage tube was located in the left renal pelvis and communicated with the outside of the body

Selection of puncture approach: The approach selected was such that it afforded the shortest path while avoiding passing through the abdominal cavity. The puncture path involved entry through the renal cortex into the renal cone and then into the dilated renal pelvis. The patient’s position was adjusted for the procedure and routine disinfection was performed. A surgical towel with a hole towel was used for coverage. After administering local anesthesia, a small incision was made in the skin, under real-time ultrasound guidance, the 7F drainage tube kit (BIOTEQ, Taiwan, China, pull-string, disposable pigtail drainage catheter with multiple side holes in the front) was inserted along the planned path into the kidney with hydronephrosis. The needle core was removed, and the outer cannula was continued to be inserted when there was urine outflow (Figs. 1, 2). The catheter was fixed and connected to the drainage bag. The patient was asked to leave until the urine from the drainage bag was observed to be clear, and the nephrostomy tube was replaced 3 months later.

### Nephrostomy tube replacement

The nephrostomy tube was first replaced 3 months after the operation. However, drainage through the nephrostomy tube was not smooth during the period, and the patients were instructed to flush the nephrostomy tube through the sterilized three-way tube by using sterile normal saline. If the drainage was still not smooth after flushing, the nephrostomy tube was replaced in a timely manner. If there is no obstruction on the surface and lumen of the replaced nephrostomy tube after 3 months, the subsequent replacement interval was appropriately extended. The nephrostomy tube was replaced by coaxial guide-wire replacement. The patient was placed in the lateral decubitus position with the affected side up, and the skin around the drainage tube and the drainage tube mouth were locally disinfected, followed by coverage with surgical towels. A smooth guide wire (the commonly used central venous catheter assembly guide wire, 16 Ga) was inserted into the original nephrostomy tube. After ultrasound-guided observation, the guide wire was inserted into the renal pelvis and was fixed, and the original nephrostomy tube was slowly retracted. The needle core of the new nephrostomy tube was pulled out (a thicker 8F or 10F drainage tube was used for nephrostomy tube replacement), and the needle sheath and outer cannula were re-inserted along the guide wire. After successful re-insertion, the needle sheath and guide wire were removed in turns. The catheter was secured and attached to the drainage bag. If the coaxial guide wire could not be used for tube replacement due to blockage or falling off of the drainage tube, the puncture approach was re-selected again, and the nephrostomy tube was placed by percutaneous puncture under ultrasound guidance.

### Observation indicators

The changes in serum creatinine and blood urea nitrogen levels before the operation and 7 days and 1 month after the operation were recorded and statistically analyzed. The success of the procedure was defined as the nephrostomy tube being correctly placed into the collecting system and the drainage being unobstructed [6]. Complications of ultrasound-guided percutaneous nephrostomy were classified according to the Percutaneous Nephrostomy Guideline of the Society of Interventional Radiology (SIR). Complications that did not require treatment or those without significant outcomes and necessitated minimal treatment were defined as minor complications. On the other hand, complications that required treatment or hospitalization for less than or longer than 48 hours or those leading to an unplanned increase in treatment or resulting in permanent sequelae or death of the patient were considered as major complications [6]. Nephrostomy tube-related complications refer to complications such as nephrostomy tube displacement, occlusion, falling off, and fracture [7]. The success rates of the puncture and complications of ultrasound-guided percutaneous nephrostomy and nephrostomy tube-related complications were recorded.

The questionnaire was used to investigate two issues: (1) whether the patients could function independently and (2) whether the patients could participate in light physical labor or normal work.

### Statistical methods

SPSS 20.0 statistical software was used for data analysis. Count data were expressed as frequencies, measurement data were expressed as mean ± standard deviation ($\overline{x}$ ± s), and paired *t*-tests were applied for mean comparison. A *P*-value of <0.05 indicated that the difference was statistically significant.

## Results

Of the 32 patients, 20 (62.5%) underwent left nephrectomy and 12 (37.5%) right nephrectomy. The patients’ pre-catheterization serum creatinine level was 323.1 ± 183.5 μmol/L, and the levels 7 days and 1 month after catheterization were 160.2 ± 72.4 and 156.5 ± 69.4 μmol/L, respectively. The patients’ pre-catheterization blood urea nitrogen level was 15.32 ± 8.93 mmol/L, and the levels 7 days and 1 month after catheterization were 9.38 ± 4.01 and 9.06 ± 3.63 mmol/L, respectively. Serum creatinine and blood urea nitrogen levels decreased significantly after the procedure. Paired t-tests showed that differences in serum creatinine and blood urea nitrogen levels between the pre-catheterization and 7-day post-catheterization and that between the pre-catheterization and 1-month post-catheterization values were significant (*P* < 0.05). There was no significant difference in serum creatinine and blood urea nitrogen levels between the 7-day and 1-month post-catheterization values (*P* > 0.05) (Table.1.)

**Table 1:** The changes of serum creatinine and blood urea nitrogen value in 32 patients before and after catheterization ($\overline{x}$ ± s)

	Pre-catheterization	7-day post-catheterization	1-month post-catheterization
Serum creatinine values (μmol/L)	323.1 ± 183.5	160.2 ± 72.4*	156.5 ± 69.4*
Blood urea nitrogen (mmol/L)	15.32 ± 8.93	9.38 ± 4.01*	9.06 ± 3.63*

*P <0.01 (compared with the pre-ablation value)

Overall, 63 ultrasound-guided percutaneous nephrostomy procedures were performed in 32 patients. The nephrostomy tube was successfully replaced using the coaxial guide wire after the first nephrostomy in 20 patients (62.5%), and the remaining 12 patients (37.5%) underwent re-puncture after the first nephrostomy because the tube fell off and was blocked. The number of re-punctures per person was 1–5. All 63 ultrasound-guided percutaneous nephrostomy procedures were successfully performed, and the puncture success rate was 100%. There was one case (3.1%) of a serious complication: perirenal hematoma after puncture, which improved after arterial embolization. Minor complications occurred in three cases (9.4%). Two patients developed gross hematuria with blood clot formation caused by bleeding at the puncture site. After clamping the nephrostomy tube and repeatedly rinsing it with sterile normal saline, the color of the urine normalized within 72 h. One patient had a postoperative fever, which resolved within 48 h after anti-infection treatment.

All patients had indwelling nephrostomy tubes for more than 12 months. The incidence of nephrostomy tube-related complications was 37.5% (12 cases), including nine cases of falling off of the nephrostomy tube and three cases of nephrostomy tube occlusion. The falling off of the tube happened accidentally because of external forces during activities owing to improper care by the patients themselves, or because they could not tolerate the discomfort caused by the nephrostomy tube and pulled it out. In such cases, the puncture approach was re-selected, and the patients underwent percutaneous nephrostomy under ultrasound guidance. For patients with nephrostomy tube occlusion, tube was found to be completely blocked by granulomatous necrotic tissue, the coaxial guide wire could not be used to replace the nephrostomy tube after repeated flushing with sterile normal saline. Hence, a new puncture approach was re-selected, and the patients underwent percutaneous nephrostomy under ultrasound guidance. None of the above patients had nephrostomy tube fracture, stenosis, and other drainage limitations caused by nephrostomy tube compression. Furthermore, they could care for the puncture point by themselves. The duration of the stoma was 56.7 ± 36.2 (range, 13–120) months, and the interval to the first tube replacement was 3.9±1.8 (range, 1–8) months. A total of 344 ostomy tube changes were performed, ranging from 4 to 24 times per person. The coaxial guide-wire method was used to replace the nephrostomy tube 310 times (90.1%). The nephrostomy tube was replaced by percutaneous re-puncture under ultrasound guidance 9 times because of nephrostomy tube shedding and 25 times because of nephrostomy tube blockage. Nephrostomy tube replacement was difficult in three of these instances; however, the tube was removed completely and safely under ultrasound guidance in all three cases; the success rate of nephrostomy tube replacement was 100%. No serious complications were noted.

All the 32 patients were treated with standard systemic anti-tuberculosis drugs. The nephrostomy tube was removed after 13–66 months after nephrostomy in 14 patients, of which 4 patients underwent ureteral stent implantation, 1 patient underwent ureteral stricture internal incision, 3 patients underwent ileal cystoplasty, and renal morphology and function returned to normal in 6 patients. Eleven patients had long-term nephrostomy until the end of the follow-up period. Five patients with persistent deterioration of renal function were treated with dialysis after removing the nephrostomy tube. One patient underwent surgical resection and conversion to dialysis treatment owing to the continuous deterioration of renal function 45 months after nephrostomy. Another patient underwent surgical resection and conversion to dialysis treatment owing to mass lesions detected on computed tomography 83 months after the nephrostomy. Twenty-six of the 32 patients could participate in light physical labor or normal work, and all patients could take care of themselves.

## Discussion

Genitourinary tuberculosis refers to tuberculosis that develops in the kidney, ureter, testis, and epididymis through blood transmission [8]. Renal tuberculosis is the most common type of genitourinary tuberculosis and develops first. It then spreads from the kidney to the whole urinary system [9]. Therefore, renal tuberculosis actually represents the significance of urinary tuberculosis [10]. Renal tuberculosis is characterized by initial invasion of the renal cortex, development in the capillary plexus of the glomerulus, often involving the formation of multiple micro-tuberculosis foci, followed by progression to the medullary junction area and the renal papilla. Its growth and reproduction in the medulla were much more active than in the cortex, resulting in renal papillary ulcers and necrosis, until spread to the renal calices, resulting in cavitary ulcers. Damage to the renal structures occurs simultaneously with fibrosis during the repair process, leading to impaired renal function and obstruction [11]. Unilateral renal tuberculosis can cause ureteral stenosis, ureteral orifice lesions, and bladder contracture to cause ureteral dilatation above the lesion, and ureteral wall thickening, stenosis, and closure of the lumen can cause renal calcification, resulting in “ autonephrectomy.” Renal tuberculosis involving the contralateral ureteral orifice can cause contralateral hydronephrosis or contralateral kidney infection.

In patients with unilateral renal tuberculosis leading to nephrectomy or resection, when the disease affects the contralateral side, the lower discharge of necrosis in the kidney combined with the thickening and adhesion of the ureteral wall often cause ureteral obstruction and hydronephrosis. In severe cases, renal function markedly deteriorates. In our patients, renal function was impaired and serum creatinine levels increased to varying degrees. The serum creatinine and blood urea nitrogen levels decreased significantly 7 days after the operation, and there was no significant difference between the serum creatinine and blood urea nitrogen levels at 1 month after the operation and 7 days after the operation, indicating that the renal function of the affected side was protected by the operation, and the operation did not affect the renal function. The basic principle of the treatment of renal tuberculosis is systemic anti-tuberculosis treatment to achieve a cure and avoid surgery. However, most patients are already in the intermediate and late stages of the disease when diagnosed clinically, with ureteral stenosis and hydronephrosis, and the function and structure of the affected kidney are damaged to varying degrees [12]. Some scholars have reported that the rate of nephrectomy in patients with renal tuberculosis is more than 50% [13]. In patients who have undergone unilateral nephrectomy, the probability of renal tuberculosis on the contralateral side leading to renal function damage and subsequent uremia is very high [14]. Hence, it is particularly important to preserve residual renal function during this time. Ultrasound-guided percutaneous nephrostomy provides an alternative, which can directly drain the hydronephrosis or empyema of the tuberculous kidney and reduce or even terminate the stimulation of the ureter and bladder by tuberculous bacteriuria or pyuria. However, if the stricture of the ureter cannot be treated in the early stage of treatment, endoluminal treatment or surgical reconstruction is needed in the later stage. Regarding the outcomes of this group of patients, all patients received systemic standardized anti-tuberculosis treatment, of whom 11 patients had nephrostomy until the end of the follow-up period, and the longest time with the tube was 120 months. The nephrostomy tube was removed in another 14 patients, and eight of them underwent urinary reconstruction surgery, which indicated that nephrostomy afforded the time required for anti-tuberculosis treatment, provided opportunities for patients to undergo urinary reconstruction surgery in the future, and reduced the rate of kidney loss. If surgical reconstruction is still not possible or the patient is too old to tolerate surgery, long-term nephrostomy can also be an option.

As a common method for resolving urinary obstruction, ultrasound-guided percutaneous nephrostomy has the advantages of simple operation and a high success rate [15, 16]. A total of 63 punctures were performed in our patient population, and the success rate was 100%. Complications after nephrostomy include bleeding, infection, pain, adjacent organ injury, and fistula obstruction or detachment [17]. The reported incidences of severe and minor complications after puncture are 0–7% and 2–38%, respectively [18]. The highest critical values of the incidences of severe and minor complications recommended by the Royal College of Radiology were 8% and 15%, respectively [19]. The incidences of major and minor complications were 3.1% (1/32) and 9.4% (3/32), respectively, which were significantly lower than the maximum values recommended by the guidelines. There was one case of a severe complication in our patient population: perirenal hematoma after the puncture that improved after arterial embolization. Among the three cases with minor complications, there were two cases of gross hematuria with blood clot formation caused by bleeding at the puncture site. After treatment, the color of urine returned to normal within 72 hours after clamping of the nephrostomy tubeand repeatedly rinsing with sterile normal saline. In our experience, the technique to improve the success rate of puncture and reduce bleeding is to choose the shortest and clearest puncture path through the renal cortex, renal cone, and renal pelvis. While inserting the needle, the direction of the puncture was adjusted to ensure that the needle was accurately inserted per the predetermined path, and rapid continuous entry into the renal pelvis was avoided. One case of severe hemorrhage in our patient group was due to patient obesity; the needle path was not clearly displayed after the fourth puncture, which was caused by accidental injury to a large blood vessel in the kidney.

The complications related to nephrostomy tube mainly include nephrostomy tube obstruction, displacement, fracture, and detachment. All patients underwent long-term nephrostomy, and 12 (37.5%) developed complications related to the nephrostomy tube. According to the literature, nephrostomy tube shedding is the most common fistula-related complication. The incidence of nephrostomy tube shedding in the early postoperative period is approximately 1%, while that after the tube has been in for 1 month is approximately 2%. The incidence can be 11–30% if the tube is in for a longer time [20]. Regarding the nine cases of the nephrostomy tube falling off in our patient group, the tube was pulled out in all cases because of improper nursing by the patients or intolerable discomfort experienced by the patients. The most common cause of early postoperative fistula obstruction was gross hematuria and blood clot formation. Flushing the fistula with normal saline helped clear the obstruction. In our patient group, three cases of complete nephrostomy tube occlusion because of a large amount of caseous necrotic tissue in the tube, caused by renal tuberculosis, were noted. The 3-month replacement of the nephrostomy tubes based on the instructions for the use of the fistula tube. Long-term retention of the nephrostomy tube will reduce the frequency of replacement and the cost to patients. In this study, the coaxial guide-wire method was used to replace the drainage tube, and the success rate was 90.1% (310/344), which was relatively high. The main reason for failure was falling off of the nephrostomy tube owing to improper patient care or blockage of the nephrostomy tube and non-feasibility of guide-wire insertion. All patients could take care of themselves during the stoma period, which improved their quality of life.

Our study has some limitations. Due to the relatively rare cases of solitary kidney with hydronephrosis due to renal tuberculosis, the number of cases in this study is relatively small, and the assessment of complications is not comprehensive enough. Meanwhile, the changes of renal function in patients with long-term catheterization still need to be observed in large samples for a long time. Another limitation is that, since this procedure was performed by experienced interventional ultrasound physician, we couldn’t evaluate the impact of experience on technical success, and complication rates.

## Conclusion

Ultrasound-guided percutaneous nephrostomy is simple and feasible and is associated with small trauma and low cost, which is conducive to the anti-tuberculosis treatment, provides an opportunity for patients to undergo reconstruction surgery in the future, reduces the rate of kidney loss, and improves patients’ quality of life. It is of great significance in the treatment and management of a solitary kidney with hydronephrosis caused by renal tuberculosis. However, the long-term effects of the procedure need to be further studied in a larger sample.
